# Evaluation of hybrid capture-based targeted and metagenomic next-generation sequencing for pathogenic microorganism detection in infectious keratitis

**DOI:** 10.1186/s12879-025-11608-9

**Published:** 2025-09-29

**Authors:** Qianqian Lan, Zhenfeng Deng, Chunhong Li, Hui Huang, Zhou Zhou, Li Jiang, Fengmei Li, Dan Wu, Min Zheng, Meihua Zhou, Qi Chen, Fan Xu

**Affiliations:** 1 Department of Ophthalmology, The People’s Hospital of Guangxi Zhuang Autonomous Region & Guangxi Key Laboratory of Eye Health & Guangxi Health Commission Key Laboratory of Ophthalmology and Related Systemic Diseases Artificial Intelligence Screening Technology & Institute of Ophthalmic Diseases, Guangxi Academy of Medical Sciences, Nanning, 530021 China; 2 Infection Diagnosis Center, Guangxi KingMed Diagnostics, Nanning, 530007 China

**Keywords:** Hybrid capture-based targeted next-generation sequencing (hc-tNGS), Metagenomic next-generation sequencing (mNGS), Infectious keratitis, Cornea, Diagnosis

## Abstract

**Objective:**

This study aimed to compare the performance of hybrid capture-based targeted next-generation sequencing (hc-tNGS) and metagenomic next-generation sequencing (mNGS) in detecting the causative pathogens of infectious keratitis.

**Methods:**

A total of 60 patients with clinically diagnosed infectious keratitis were enrolled between January and December 2024. Corneal scraping samples were analyzed using hc-tNGS and mNGS. Detection rates, pathogen spectra, normalized reads, turnaround time (TAT), and costs were compared between the two techniques.

**Results:**

hc-tNGS exhibited a significantly higher overall detection rate than mNGS (86.7% versus 73.3%, *P* < 0.001). In particular, hc-tNGS detected 29 pathogens (13 bacteria, 9 viruses, and 7 fungi), whereas mNGS detected 22 pathogens (9 bacteria, 7 viruses, and 6 fungi). Furthermore, hc-tNGS detected additional low-abundance pathogens in 17 mNGS-positive patients (28.3%, 17/60) and 8 mNGS-negative patients (11.3%, 8/60). The normalized reads for viruses, bacteria, and fungi in hc-tNGS were 57.2-, 2.7-, and 3.3-fold higher than those in mNGS, respectively (*P* < 0.001,* P* = 0.003, and* P* = 0.028). Moreover, hc-tNGS reduced TAT by 11.3% (18.0 versus 20.3 h) and costs by 22.4–48.8%. The median of sequencing data size of mNGS was 29.8 million (29.8 M) reads, which was significantly higher than that of tNGS (1.5 M, *P* < 0.001).

**Conclusion:**

hc-tNGS demonstrates superior performance and cost-effectiveness in detecting potential pathogens of infectious keratitis, especially low-abundance pathogens, whereas mNGS remains valuable for detecting novel pathogens. Owing to its enhanced performance, faster TAT, and reduced costs, hc-tNGS is a promising clinical tool for pathogen detection.

**Supplementary Information:**

The online version contains supplementary material available at 10.1186/s12879-025-11608-9.

## Introduction

Infectious keratitis is a vision-threatening ophthalmic disease that requires prompt and accurate identification of pathogens for effective treatment [1]. Traditional microbial culture is not suitable for rapid pathogen detection owing to limitations such as a prolonged turnaround time (TAT), low sensitivity, and high rates of false-positive and false-negative results, particularly in the detection of fastidious microorganisms [2]. Immunological assays and polymerase chain reaction (PCR) yield rapid and precise results, as they do not require microbial culture. However, they necessitate a previously established etiological hypothesis and exhibit narrow detection ranges, which limit their ability to detect diverse pathogens [3]. Furthermore, the wide variety of pathogens responsible for causing infectious keratitis; the widespread use of antibiotics, corticosteroids, and immunosuppressants; and the prevalence of comorbidities such as transplantation, malignancies, and immunodeficiency collectively challenge the rapid and accurate identification of causative pathogens in clinical settings [4]. In recent years, metagenomic next-generation sequencing (mNGS) has demonstrated potential in the diagnosis of infectious diseases, including infectious keratitis [5].

As an unbiased sequencing technology, mNGS can detect known, unknown, and even unexpected pathogens, showing remarkable promise in diagnosing infectious keratitis, particularly in culture-negative cases [6]. Recent studies have validated the utility of mNGS in pathogen detection in infectious keratitis, highlighting the broader applicability of NGS in diagnosing ocular infections [7–9]. However, the widespread application of mNGS is limited by high costs, small volumes of ocular samples, and interference from host nucleic acids [5]. On the contrary, hybrid capture-based targeted next-generation sequencing (hc-tNGS) provides a more focused approach to pathogen detection, as it uses pathogen-specific probes to enrich target sequences. This method enhances sequencing efficiency, sensitivity, and cost-effectiveness when specific pathogens or pathogen groups are suspected.

In this study, we used hc-tNGS to detect more than 20,000 microbial species in clinical ocular samples and compared its diagnostic value with that of mNGS in infectious keratitis.

## Methods

### Study population

Patients with clinically diagnosed infectious keratitis were enrolled from The People's Hospital of Guangxi Zhuang Autonomous Region between January and December 2024. Ocular samples from these patients were analyzed using hc-tNGS and mNGS. This study was approved by the local ethics committee (approval number: KY-KJT-2024–163), and all data used in this study were obtained anonymously and used exclusively for analysis. The confidentiality of patient information was rigorously maintained, and written informed consent was obtained from all patients.

The inclusion criteria were as follows: (1) age ≥ 18 years; (2) diagnosis of infectious keratitis based on the presence of corneal ulcers in the affected eye (with or without hypopyons), positive corneal fluorescein staining, and history/signs of infection; and (3) willingness to participate in the study and sign the informed consent form.

The exclusion criteria were as follows: (1) concurrent severe systemic diseases compromising participation in the study; (2) ongoing pregnancy or lactation; and (3) inability to cooperate with sample collection.

### Sample collection and preparation

Corneal scraping samples were collected from all patients, stored at −20 °C, and transported to the laboratory within 24 h. Before nucleic acid extraction, the samples were mixed with 600 μL of ddH_2_O on a vortex, and 300-μL aliquots were transferred to two microcentrifuge tubes for hc-tNGS and mNGS.

### Workflow of hc-tNGS

**Fig. 1:**
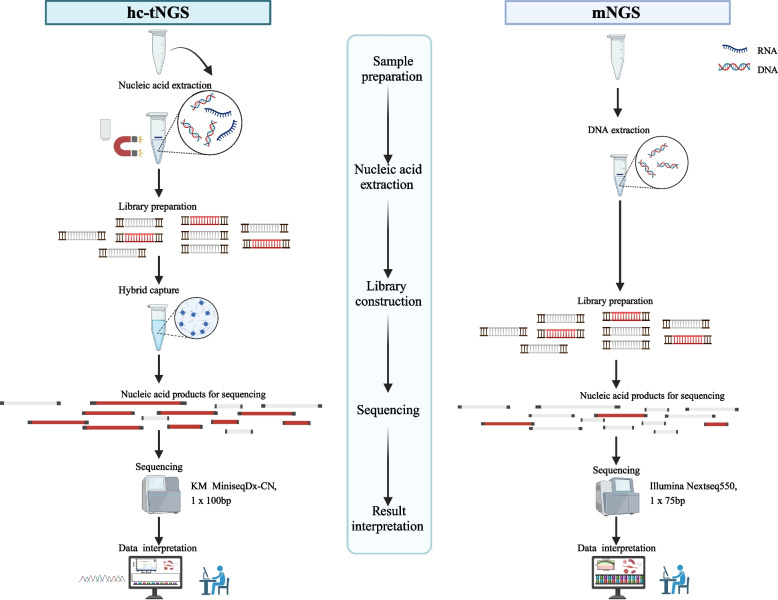
The workflow of hc-tNGS and mNGS

hc-tNGS was performed as described in our previous study [10]. The workflow is shown in Fig. 1.

#### Nucleic acid extraction

DNA and RNA were extracted from the 300-μL aliquots using the MagPure Pathogen DNA/RNA Extraction Kit (Magen Biotechnology, Guangzhou, China). A negative control and a positive control were included in each batch to monitor the hc-tNGS process.

#### Library construction and sequencing

cDNA was synthesized using a reverse transcription kit (KingCreate, Guangzhou, China). Libraries were constructed from the synthesized cDNA using a library construction kit (KingCreate, Guangzhou, China). Eight uniquely barcoded libraries were pooled and hybridized with specific biotinylated probes for 0.5 h using the MetaCAP™ Pathogen Capture Metagenomic Assay Kit (KingCreate, Guangzhou, China). The quality and quantity of the constructed libraries were evaluated using the Qsep100 Bio-Fragment Analyzer (Bioptic, Taiwan, China) and the Qubit 4.0 fluorometer (Thermo Fisher Scientific, MA, USA), respectively. Subsequently, the diluted and denatured libraries were sequenced on an KM MiniseqDx-CN platform using a universal sequencing reagent kit (KS107-CXR, KingCreate, Guangzhou, China). On average, each library yielded approximately 1 million (1 M) reads, with a single-end sequencing read length of 100 bp.

#### Bioinformatic analysis

Fastp (version 0.23.1) was used to remove adapters, low-quality reads, reads with > 5 N bases, and reads with a length of 35 bp to obtain clean reads [11]. These reads were aligned to the human reference genome (hg38) using Burrows–Wheeler Aligner (BWA; version 0.7.17r1188), and human reads were filtered [12]. For taxonomic classification, the remaining reads were compared to a reference database containing 21,388 microbial species, which included 11,958 bacteria; 7373 viruses (4,414 RNA viruses and 2,959 DNA viruses); 1714 fungi; and 343 parasites. The number of reads per million sequencing reads (RPM) was calculated at the species and genus levels.

### Workflow of mNGS

mNGS was performed as described in our previous study [13]. The workflow is shown in Fig. 1.

#### Nucleic acid extraction

DNA was extracted from the 300-μL aliquots using the Universal DNA/RNA Extraction Kit (TR202JY-50, Jianshi, Beijing, China). A negative control and a positive control were included in each batch to monitor the mNGS process.

#### Library construction and sequencing

After the extracted DNA was quantified, libraries were constructed using the Pathogenic Metagenomic DNA Detection Kit (KS619-DNAmN48, KingCreate, Guangzhou, China). The library construction process involved end repair, adapter ligation, magnetic bead-based purification, and PCR. The quality and quantity of the constructed libraries were evaluated using the Qsep100 Bio-Fragment Analyzer (Bioptic, Taiwan, China) and the Qubit 4.0 fluorometer (Thermo Fisher Scientific, MA, USA), respectively. The library fragments exhibited sizes ranging from 250 to 350 bp. After the concentration of the pooled library was reassessed, it was diluted to a final concentration of 2 nmol/L. Subsequently, 10 µL of the pooled library was mixed with 10 µL of freshly prepared NaOH (0.1 mol/L) on a vortex. The mixture was centrifuged, and the resulting library was incubated at room temperature for 5 min. Single-end 50-bp sequencing was performed on the Illumina NextSeq 550 System using the 75-cycle Reagent Kit (20024906, Illumina, CA, USA). Each library produced more than 10 M of data.

#### Bioinformatic analysis

Adapters, short reads, and low-quality reads were removed from raw data using Fastp (version 0.23.1) [11]. Low-complexity and duplicate reads were removed using the DUST and Bloom filter algorithms with an in-house program [14]. Reads with single-end lengths exceeding 35 bp that had a Q30 value of > 75% were retained to obtain high-quality data. These reads were aligned to the human reference genome (hg38) using BWA (version 0.7.17-r1188) [12]. Finally, The unmapped reads were compared to the same reference database used for taxonomic classification in hc-tNGS. The reference sequences used for mapping were curated from various sources, including the GenBank, RefSeq, and Nucleotide databases from the National Center for Biotechnology Information (NCBI, https://www.ncbi.nlm.nih.gov/).

### Interpretation of the results of hc-tNGS and mNGS

The performance of hc-tNGS and mNGS was evaluated based on normalized reads, the negative control ratio (NCR), and clinical relevance. Normalized reads were defined as the RPM for specific microbial sequences. To minimize the risk of contamination, a true-positive result was considered when the NCR of a species or genus was ≥ 10. The NCR was defined as the RPM for specific microbial sequences detected in samples being at least tenfold higher than that for the same microbial sequence in the negative control.

### Statistical analysis

Quantitative variables were expressed as medians with accompanying ranges, whereas categorical variables were expressed as counts with percentages. All statistical analyses were performed using the SPSS (version 25.0) software (IBM, Armonk, NY, USA.). The chi-square test was used to compare the detection rates of hc-tNGS and mNGS. The Kappa test was used to assess the agreement between the two methods. A *P*-value of < 0.05 was considered statistically significant.

## Results

### Patient characteristics

A total of 60 patients (60 eyes) with infectious keratitis were included in this study. This patient cohort included 35 men (58.3%) and 25 women (41.7%) with a median age of 57.5 years (interquartile range [IQR], 50–68 years).

### Detection performance

**Fig. 2:**
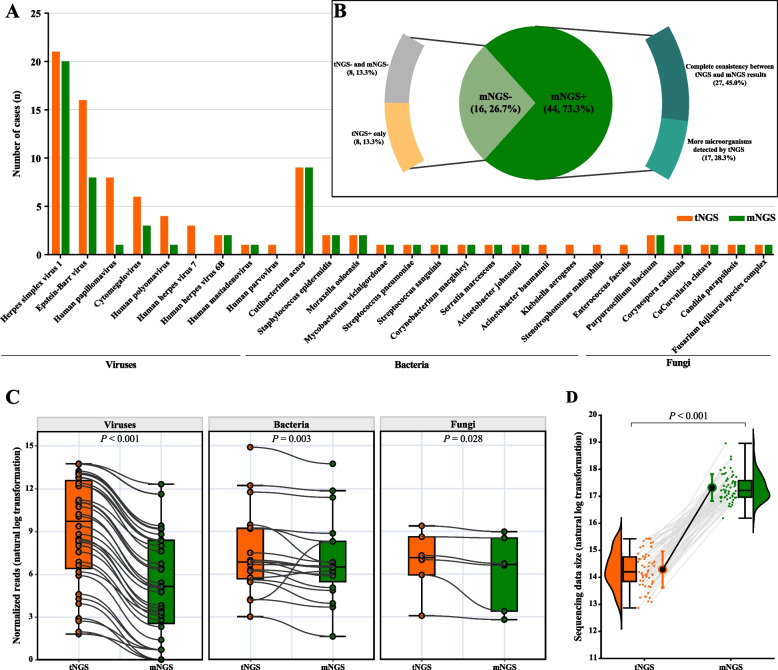
Comparison of the detection performance of hc-tNGS and mNGS. **A** Distribution of pathogens in the study cohort and the respective contributions of hc-tNGS and mNGS to their detection. **B** Detection results of hc-tNGS and mNGS. **C** Normalization and comparison of the reads of viruses, bacteria, and fungi detected by hc-tNGS and mNGS. **D** Comparison of the sequencing data size between hc-tNGS and mNGS

The overall microbial detection rate of hc-tNGS (86.7%, 52/60 patients) was significantly higher than that of mNGS (73.3%, 44/60 patients) (*P* < 0.001). hc-tNGS detected 29 microbial species (13 bacteria, 9 viruses, and 7 fungi), whereas mNGS detected 22 microbial species (9 bacteria, 7 viruses, and 6 fungi). Detailed results are shown in Fig. 2A and B and Supplementary Table 1.

hc-tNGS yielded positive findings in the 44 pathogen-positive patients detected via mNGS (73.3%, 44/60). The two techniques showed complete concordance in pathogen identification in 27 of these patients (45.0%, 27/60), detecting 12 cases of herpes simplex virus type 1 (HSV-1) infection; 6 cases of Epstein–Barr virus (EBV) infection; 6 cases of *Cutibacterium acnes* infection; 2 cases of cytomegalovirus (CMV) infection; and 1 case each of human polyomavirus (HPyVs), human papillomavirus (HPV) type 14 (HPV-14), human herpesvirus 6B (HHV-6B), *Streptococcus sanguinis*, *Mycobacterium vicinigordonae*, *Moraxella osloensis*, *Streptococcus pneumoniae*, *Fusarium fujikuroi* species complex, and *Purpureocillium lilacinum* infection (Supplementary Table 2). In the remaining 17 patients (28.3%, 17/60), hc-tNGS detected additional potential pathogens, identifying 8 cases of EBV infection; 7 cases of HPV infection; 3 cases of CMV infection; 3 cases of HPyVs infection; and 1 case each of HSV-1, human parvovirus B19, *Acinetobacter baumannii*, *Stenotrophomonas maltophilia*, *Enterococcus faecalis*, *Klebsiella aerogenes*, *Malassezia restricta*, and *M. globosa* infection (Supplementary Table 3).

Among the 8 patients (13.3%, 8/60) with negative mNGS results, hc-tNGS identified 5 cases of EBV infection; 2 cases of HPV infection; and 1 case each of CMV, human herpesvirus 7 (HHV-7), human parvovirus B19, and *M. restricta* infection.

Both hc-tNGS and mNGS yielded negative results in 8 patients (13.3%, 8/60).

### Comparison of pathogen spectra

Bacterial, fungal, and viral pathogens were detected in 6 (10.0%, 6/60), 2 (3.3%, 2/60), and 23 (38.3%, 23/60) patients, respectively. With regard to coinfection, bacterial–fungal coinfection was detected in 2 (3.3%, 2/60) patients, bacterial–viral coinfection was detected in 5 patients (8.3%, 5/60), viral–fungal coinfection was detected in 11 patients (18.3%, 11/60), and bacterial–fungal–viral coinfection was detected in 3 patients (5.0%, 3/60). The types and frequencies of pathogens detected by hc-tNGS and mNGS are summarized in Supplementary Table 4. Compared to mNGS, hc-tNGS demonstrates a significantly higher proportion of viral detection (70.0% vs. 46.7%, *P* < 0.001), primarily observed in EBV and HPV.

### Comparison of detected reads, TAT, sequencing data size, and costs

When detecting identical pathogens, hc-tNGS demonstrated significantly higher normalized reads for viruses, bacteria, and fungi compared with mNGS, with mean fold ratios (hc-tNGS/mNGS) of 57.2, 2.7, and 3.3, respectively (*P* < 0.001, *P* = 0.003, and *P* = 0.028; Fig. 2C). hc-tNGS demonstrated a mean laboratory TAT of 18.0 h as opposed to 20.3 h required by mNGS, representing an 11.3% reduction in processing time. The median of sequencing data size of mNGS was 29.8 M reads (IQR: 23.1–44.1 M), which was significantly higher than that of tNGS (1.5 M, IQR: 1.0–2.6 M, *P* < 0.001, Fig. 2D). The detection costs of hc-tNGS were 22.4–48.8% lower than those of mNGS.

## Discussion

When conventional tests fail to meet the laboratory diagnostic requirements, mNGS can be used to analyze ocular samples. The unbiased nature of this technology makes it a crucial tool for identifying unknown or unexpected pathogens [15, 16]. A study showed that mNGS had a sensitivity of 88% in diagnosing endophthalmitis and that its sensitivity in detecting unknown, low-abundance microorganisms exceeded that of other methods [17]. Another similar study reported that the pathogen detection rate of mNGS in infectious keratitis was 92.7%, which was higher than the 69.3% detection rate achieved without the use of mNGS [8]. However, the accuracy and specificity of mNGS warrant further clinical validation, as they may be influenced by sample volume, specimen processing, nucleic acid extraction, library preparation, and sequencing depth. To date, the application value of hc-tNGS in infectious keratitis remains unclear. In this study, we compared the performance of hc-tNGS and mNGS in diagnosing infectious keratitis. The findings highlight the unique strengths and clinical implications of each method, providing valuable insights into the diagnosis of ocular infections.

Compared with mNGS (73.3%), hc-tNGS demonstrated a high detection rate (86.7%), particularly in detecting low-abundance pathogens. This enhanced performance may be attributed to the probe-based enrichment strategy of hc-tNGS, which amplifies target microbial sequences while reducing interference from host DNA [18]. Notably, hc-tNGS identified 29 microbial species, including common latent viruses such as EBV and HPV that were not detected by mNGS. On the contrary, mNGS identified 22 microbial species. Although hc-tNGS is a targeted method, the use of millions of homologous probes enables it to achieve a detection rate comparable to that of mNGS and identify unexpected pathogens. In this study, both hc-tNGS and mNGS identified *M. vicinigordonae*, an extremely rare non-tuberculous mycobacterium. The higher viral detection rate of hc-tNGS (38.3% viral-only infections identified in this study) may help reassess culture-negative cases and guide antiviral therapy. In addition, the unbiased nature of mNGS is crucial for detecting unknown pathogens.

The operational benefits of hc-tNGS are considerable. With an 11.3% faster laboratory TAT (18.0 versus 20.3 h) and 22.4–48.8% lower costs, hc-tNGS addresses two limitations to the widespread clinical application of mNGS. These enhanced features are attributed to the streamlined workflow of hc-tNGS: probe enrichment does not require the detection of host nucleic acids, and optimized bioinformatic pipelines minimize the calculation and cost burdens. Furthermore, hc-tNGS showed higher normalized reads for viruses (57.2-fold), bacteria (2.7-fold), and fungi (3.3-fold), enhancing confidence in the detection of true-positive results. This enhanced sequencing depth is particularly important for interpreting pathogens such as intracellular bacteria and thick-walled fungi, which have low nucleic-acid-extraction efficiency and encounter interference from a majority of host nucleic acids in mNGS. The strength of mNGS lies in its ability to complement the detection of novel species [15, 19, 20]. hc-tNGS may serve as a promising tool for pathogen detection in resource-limited settings owing to its cost-effectiveness and enhanced performance, whereas mNGS remains indispensable for research and epidemiological surveillance.

Accurate pathogen identification via hc-tNGS and mNGS has profound implications for antimicrobial stewardship. In this study, mixed infections, predominantly bacterial–viral coinfection, were detected in 28.3% of the patients. Conventional methods frequently fail to detect these infections. Despite their specific advantages, both hc-tNGS and mNGS encounter challenges with regard to result interpretation. For instance, the recognition of false-positive results requires careful correlation with clinical findings, such as detecting latent viruses (e.g., EBV, CMV, HPyVs, HPV, HHV-7, and HHV-6B) of uncertain clinical significance [21–23]. Similarly, detection of skin-colonizing bacteria or environmental contaminants (e.g., *C. acnes*, *S. epidermidis*, and *M. osloensis*) necessitates stringent controls [24, 25], which is highlighted by the NCR criterion (NCR ≥ 10) defined in this study. Standardization remains a critical challenge, as variations in probe design, sequencing platforms, and bioinformatic analysis complicate inter-laboratory comparisons. Moreover, neither method can distinguish between viable and non-viable organisms, a limitation shared with molecular identification techniques. In the future, unified guidelines for probe selection, contamination thresholds, and result interpretation should be established to ensure clinical reliability.

The clinical application of mNGS in diagnosing ocular infections relies on addressing its existing limitations. Expanding probe libraries to include emerging pathogens and resistance markers may enhance the versatility of hc-tNGS. This study has several limitations that warrant consideration. First, all patients were enrolled from a single hospital, which may limit the generalizability of the results. Therefore, multicenter studies are required to validate the results of this study. Second, although the sample size was determined based on statistical calculations, increasing it may enhance the statistical power of the study. Third, this study did not include samples from non-keratitis patients as controls, limiting the interpretation of results in the context of the ocular microbiome. In fact, current research on the ocular microbiome remains scarce, and understanding of microbial ecology in this specialized site lags behind more extensively studied areas such as the microbiome of gut, skin, or urogenital tract. Future studies establishing the ocular microbiome baseline in healthy populations could improve the accuracy of interpreting such molecular identification techniques. Fourth, we primarily focused on the diagnostic performance of the two techniques and did not include long-term follow-up. Future studies should focus on investigating treatment efficacy and long-term prognosis under hc-tNGS-guided therapy.

In conclusion, hc-tNGS and mNGS offer distinct advantages in the diagnosis of infectious keratitis. hc-tNGS can detect low-abundance pathogens owing to its probe-based enrichment strategy, which mitigates interference from host DNA and enhances sensitivity. This targeted approach, combined with reduced TAT and costs, makes hc-tNGS a promising tool for pathogen detection in resource-limited settings, where rapid, cost-effective diagnosis is paramount. The indispensable role of mNGS in identifying novel pathogens emphasizes its value in epidemiological surveillance and exploratory research. However, in both methods, challenges persist in standardizing the result interpretation criteria and distinguishing pathogenic from colonizing or latent microorganisms. With continuous development, the diagnostic accuracy of hc-tNGS and mNGS may improve, enhancing the understanding of ocular microbiome dynamics under both physiological and pathological conditions.

## Supplementary Information

Supplementary Material 1: Table 1. Comparison of the performance of hc-tNGS and mNGS

Supplementary Material 2: Table 2. Concordant results between hc-tNGS and mNGS

Supplementary Material 3: Table 3. Additional microorganisms detected by hc-tNGS

Supplementary Material 4: Table 4. Types and frequencies of pathogens detected by hc-tNGS and mNGS

## Data Availability

The study data is provided within the manuscript or supplementary information files. The raw sequencing data of hc-tNGS and mNGS can be found at: https://www.ncbi.nlm.nih.gov/bioproject/PRJNA1279828.
