# Supplementary material for: Evaluation of hybrid capture-based targeted and metagenomic next-generation sequencing for pathogenic microorganism detection in infectious keratitis
Source: BMC Infect Dis. 2025 Sep 29;25:1211. doi: 10.1186/s12879-025-11608-9 (PMC12482119; doi:10.1186/s12879-025-11608-9)
Supplement: Supplementary file 1 — Supplementary Material 1: Table 1. Comparison of the performance of hc-tNGS and mNGS [file 12879_2025_11608_MOESM1_ESM.docx]

**Supplementary Table 1. Comparison of the performance of hc-tNGS and mNGS.**

| hc-tNGS | mNGS | | Total |
| --- | --- | --- | --- |
|  | Positive | Negative |  |
| Positive | 44 | 8 | 52 |
| Negative | 0 | 8 | 8 |
| Total | 44 | 16 | 60 |

Positive percent agreement = 100% (95% CI: 0.900-1.000)

Negative percent agreement = 50.0% (95% CI: 0.255-0.745)
