# Supplementary material for: Evaluation of hybrid capture-based targeted and metagenomic next-generation sequencing for pathogenic microorganism detection in infectious keratitis
Source: BMC Infect Dis. 2025 Sep 29;25:1211. doi: 10.1186/s12879-025-11608-9 (PMC12482119; doi:10.1186/s12879-025-11608-9)
Supplement: Supplementary file 2 — Supplementary Material 2: Table 2. Concordant results between hc-tNGS and mNGS [file 12879_2025_11608_MOESM2_ESM.docx]

**Supplementary Table 2. Concordant results between hc-tNGS and mNGS.**

| **Patient numbers** | **tNGS results (reads)** | **mNGS results (reads)** |
| --- | --- | --- |
| P01 | Herpes simplex virus 1 (11694) | Herpes simplex virus 1 (151) |
| P05 | Herpes simplex virus 1 (901771) | Herpes simplex virus 1 (110191) |
| P08 | Cutibacterium acnes (1057) | Cutibacterium acnes (652) |
| P09 | Herpes simplex virus 1 (22976) | Herpes simplex virus 1 (219) |
| P10 | Malassezia restricta (111) | Malassezia restricta (91) |
| P11 | Cutibacterium acnes (536) | Cutibacterium acnes (388) |
| P13 | Epstein-Barr virus (45664) | Epstein-Barr virus (561) |
| P14 | Epstein-Barr virus (4710) | Epstein-Barr virus (37) |
| P15 | Cutibacterium acnes (67), Fusarium fujikuroi species complex (21) | Cutibacterium acnes (39), Fusarium fujikuroi species complex (16) |
| P18 | Purpureocillium lilacinum (5374), Epstein-Barr virus (98), Cytomegalovirus (18) | Purpureocillium lilacinum (4931), Epstein-Barr virus (2), Cytomegalovirus (1) |
| P19 | Herpes simplex virus 1 (60680), Epstein-Barr virus (6) | Herpes simplex virus 1 (624), Epstein-Barr virus (2) |
| P20 | Cutibacterium acnes (929), Human polyomavirus 5 (1899) | Cutibacterium acnes (348), Human polyomavirus 5 (30) |
| P22 | Malassezia restricta (17) | Malassezia restricta (103) |
| P23 | Streptococcus sanguinis (138191) | Streptococcus sanguinis (199075) |
| P24 | Herpes simplex virus 1 (195814) | Herpes simplex virus 1 (4248) |
| P25 | Human papillomavirus type 14 (471), Malassezia restricta (93) | Human papillomavirus type 14 (27), Malassezia restricta (289) |
| P27 | Mycobacterium vicinigordonae (125393) | Mycobacterium vicinigordonae (86162) |
| P28 | Herpes simplex virus 1 (3778) | Herpes simplex virus 1 (45) |
| P30 | Herpes simplex virus 1 (27036) | Herpes simplex virus 1 (173) |
| P33 | Cytomegalovirus (920909), Malassezia restricta (23) | Cytomegalovirus (112016), Malassezia restricta (501) |
| P37 | Herpes simplex virus 1 (6636), Epstein-Barr virus (363) | Herpes simplex virus 1 (166), Epstein-Barr virus (4) |
| P41 | Herpes simplex virus 1 (347806) | Herpes simplex virus 1 (6602) |
| P43 | Herpes simplex virus 1 (235388) | Herpes simplex virus 1 (4240) |
| P49 | Cutibacterium acnes (1767), Moraxella osloensis (775), Acinetobacter johnsonii (943), Malassezia restricta (116) | Cutibacterium acnes (926), Moraxella osloensis (707), Acinetobacter johnsonii (633), Malassezia restricta (128) |
| P52 | Cutibacterium acnes (308), Herpes simplex virus 1 (443565), Human herpes virus-6B (7), Malassezia restricta (19) | Cutibacterium acnes (484), Herpes simplex virus 1 (9860), Human herpes virus-6B (1), Malassezia restricta (40) |
| P57 | Streptococcus pneumoniae (915182) | Streptococcus pneumoniae (2922089) |
| P58 | Epstein-Barr virus (2974), Herpes simplex virus 1 (951) | Epstein-Barr virus (22), Herpes simplex virus 1 (16) |
