# Supplementary material for: Evaluation of hybrid capture-based targeted and metagenomic next-generation sequencing for pathogenic microorganism detection in infectious keratitis
Source: BMC Infect Dis. 2025 Sep 29;25:1211. doi: 10.1186/s12879-025-11608-9 (PMC12482119; doi:10.1186/s12879-025-11608-9)
Supplement: Supplementary file 3 — Supplementary Material 3: Table 3. Additional microorganisms detected by hc-tNGS [file 12879_2025_11608_MOESM3_ESM.docx]

**Supplementary Table 3. Additional microorganisms detected by hc-tNGS.**

| **Patient numbers** | **tNGS results (reads)** | **mNGS results (reads)** | **Additional microorganisms detected by hc-tNGS** | **Numbers of additional microorganisms** |
| --- | --- | --- | --- | --- |
| P03 | Herpes simplex virus 1 (198367), Human herpes virus-6B (50), Human herpes virus-7 (9), Cytomegalovirus (8) | Herpes simplex virus 1 (2019), Human herpes virus-6B (1) | Human herpes virus-7, Cytomegalovirus | 2 |
| P04 | Acinetobacter baumannii (55), Herpes simplex virus 1 (555525), Human mastadenovirus D (15) | Herpes simplex virus 1 (10067), Human mastadenovirus D (2) | Acinetobacter baumannii | 1 |
| P06 | Epstein-Barr virus (32), Malassezia restricta (34) | Malassezia restricta (58) | Epstein-Barr virus | 1 |
| P07 | Epstein-Barr virus (1691) | Negative | Epstein-Barr virus | 1 |
| P12 | Epstein-Barr virus (14) | Negative | Epstein-Barr virus | 1 |
| P16 | Purpureocillium lilacinum (1072), Epstein-Barr virus (71), Cytomegalovirus (10) | Purpureocillium lilacinum (722), Epstein-Barr virus (2) | Cytomegalovirus | 1 |
| P21 | Cutibacterium acnes (126), Human polyomavirus 5 (93), Moraxella osloensis (227) | Cutibacterium acnes (265), Moraxella osloensis (51) | Human polyomavirus 5 | 1 |
| P29 | Candida parapsilosis (368), Human papillomavirus type 41 (3), Epstein-Barr virus (28) | Candida parapsilosis (29) | Human papillomavirus type 41, Epstein-Barr virus | 2 |
| P31 | Herpes simplex virus 1 (357959), Human papillomavirus type 41 (6) | Herpes simplex virus 1 (4432) | Human papillomavirus type 41 | 1 |
| P32 | Serratia marcescens (10209), Herpes simplex virus 1 (62262), Epstein-Barr virus (22) | Serratia marcescens (3926), Herpes simplex virus 1 (837) | Epstein-Barr virus | 1 |
| P34 | Cutibacterium acnes (63), Human polyomavirus 5 (8), Herpes simplex virus 1 (933970), Malassezia restricta (4) | Cutibacterium acnes (3934), Herpes simplex virus 1 (222525), Malassezia restricta (312) | Human polyomavirus 5 | 1 |
| P35 | Herpes simplex virus 1 (235), Malassezia restricta (76) | Malassezia restricta (63) | Herpes simplex virus 1 | 1 |
| P36 | Epstein-Barr virus (46) | Negative | Epstein-Barr virus | 1 |
| P38 | Epstein-Barr virus (749), Malassezia globosa (13) | Epstein-Barr virus (10) | Malassezia globosa | 1 |
| P39 | Epstein-Barr virus (32) | Negative | Epstein-Barr virus | 1 |
| P40 | Epstein-Barr virus (26), Cytomegalovirus (45) | Negative | Epstein-Barr virus, Cytomegalovirus | 2 |
| P44 | Human papillomavirus type 41 (8), Malassezia restricta (47), Human herpes virus-7 (18) | Negative | Human papillomavirus type 41, Malassezia restricta, Human herpes virus-7 | 3 |
| P45 | Corynebacterium macginleyi (9520), Staphylococcus epidermidis (494), Herpes simplex virus 1 (485308), Human herpes virus-7 (10) | Corynebacterium macginleyi (6930), Staphylococcus epidermidis (154), Herpes simplex virus 1 (12171) | Human herpes virus-7 | 1 |
| P46 | Curvularia clatava (7674), Human polyomavirus 6 (17) | Curvularia clatava (11559) | Human polyomavirus 6 | 1 |
| P47 | Herpes simplex virus 1 (473986), Cytomegalovirus (4191), Human papillomavirus type 41 (5) | Herpes simplex virus 1 (9490), Cytomegalovirus (115) | Human papillomavirus type 41 | 1 |
| P48 | Stenotrophomonas maltophilia (37), Staphylococcus epidermidis (20), Enterococcus faecalis (17), Klebsiella aerogenes (12) | Staphylococcus epidermidis (5) | Stenotrophomonas maltophilia, Enterococcus faecalis, Klebsiella aerogenes | 3 |
| P51 | Cutibacterium acnes (12639), Herpes simplex virus 1 (73376), Human papillomavirus type 16 (118), Malassezia restricta (362) | Cutibacterium acnes (895), Herpes simplex virus 1 (2980), Malassezia restricta (366) | Human papillomavirus type 16 | 1 |
| P53 | Human papillomavirus type 41 (70) | Negative | Human papillomavirus type 41 | 1 |
| P56 | Corynespora cassiicola (815), Human papillomavirus 107 (7) | Corynespora cassiicola (1455) | Human papillomavirus 107 | 1 |
| P60 | Human parvovirus B19 (114) | Negative | Human parvovirus B19 | 1 |
