# Supplementary material for: Evaluation of hybrid capture-based targeted and metagenomic next-generation sequencing for pathogenic microorganism detection in infectious keratitis
Source: BMC Infect Dis. 2025 Sep 29;25:1211. doi: 10.1186/s12879-025-11608-9 (PMC12482119; doi:10.1186/s12879-025-11608-9)
Supplement: Supplementary file 4 — Supplementary Material 4: Table 4. Types and frequencies of pathogens detected by hc-tNGS and mNGS [file 12879_2025_11608_MOESM4_ESM.docx]

**Supplementary Table 4. Types and frequencies of pathogens detected by hc-tNGS and mNGS.**

| **Pathogens** | **hc-tNGS** | | **mNGS** | | ***P* value from χ^2^/Fisher test** | **Kappa value** |
| --- | --- | --- | --- | --- | --- | --- |
|  | **Frequencies** | **Percentage** | **Frequencies** | **Percentage** |  |  |
| **Virus** | 42 | 70.0% | 28 | 46.7% | **<0.001** | 0.545 |
| herpes simplex virus type 1 | 21 | 35.0% | 20 | 33.3% | 1.000 | 0.963 |
| Epstein-Barr virus | 16 | 26.7% | 8 | 13.3% | **0.008** | 0.595 |
| human papillomavirus | 8 | 13.3% | 1 | 1.7% | **0.016** | 0.198 |
| cytomegalovirus | 6 | 10.0% | 3 | 5.0% | 0.250 | 0.643 |
| human polyomavirus | 4 | 6.7% | 1 | 1.7% | 0.250 | 0.384 |
| human herpesvirus 7 | 3 | 5.0% | 0 | 0.0% | / | / |
| human herpesvirus 6B | 2 | 3.3% | 2 | 3.3% | 1.000 | 1.000 |
| Human adenovirus | 1 | 1.7% | 1 | 1.7% | 1.000 | 1.000 |
| human parvovirus B19 | 1 | 1.7% | 0 | 0.0% | / | / |
| **Bacteria** | 16 | 26.7% | 14 | 23.3% | 0.500 | 0.911 |
| *Cutibacterium acnes* | 9 | 15.0% | 9 | 15.0% | 1.000 | 1.000 |
| *Staphylococcus epidermidis* | 2 | 3.3% | 2 | 3.3% | 1.000 | 1.000 |
| *Moraxella osloensis* | 2 | 3.3% | 2 | 3.3% | 1.000 | 1.000 |
| *Mycobacterium vicinigordonae* | 1 | 1.7% | 1 | 1.7% | 1.000 | 1.000 |
| *Streptococcus pneumoniae* | 1 | 1.7% | 1 | 1.7% | 1.000 | 1.000 |
| *Streptococcus sanguinis* | 1 | 1.7% | 1 | 1.7% | 1.000 | 1.000 |
| *Corynebacterium macginleyi* | 1 | 1.7% | 1 | 1.7% | 1.000 | 1.000 |
| *Serratia marcescens* | 1 | 1.7% | 1 | 1.7% | 1.000 | 1.000 |
| *Acinetobacter johnsonii* | 1 | 1.7% | 1 | 1.7% | 1.000 | 1.000 |
| *Acinetobacter baumannii* | 1 | 1.7% | 0 | 0.0% | / | / |
| *Klebsiella aerogenes* | 1 | 1.7% | 0 | 0.0% | / | / |
| *Stenotrophomonas maltophilia* | 1 | 1.7% | 0 | 0.0% | / | / |
| *Enterococcus faecalis* | 1 | 1.7% | 0 | 0.0% | / | / |
| **Fungi** | 18 | 30.0% | 16 | 26.7% | 0.500 | 0.918 |
| *Malassezia restricta* | 11 | 18.3% | 10 | 16.7% | 1.000 | 0.942 |
| *Purpureocillium lilacinum* | 2 | 3.3% | 2 | 3.3% | 1.000 | 1.000 |
| *Malassezia globosa* | 1 | 1.7% | 0 | 0.0% | / | / |
| *Corynespora cassiicola* | 1 | 1.7% | 1 | 1.7% | 1.000 | 1.000 |
| *Curvularia clatava* | 1 | 1.7% | 1 | 1.7% | 1.000 | 1.000 |
| *Candida parapsilosis* | 1 | 1.7% | 1 | 1.7% | 1.000 | 1.000 |
| *Fusarium fujikuroi species complex* | 1 | 1.7% | 1 | 1.7% | 1.000 | 1.000 |
